# The contribution of multifocal visual evoked potentials in patients with optic neuritis and multiple sclerosis: a review

**DOI:** 10.1007/s10633-020-09799-4

**Published:** 2020-12-31

**Authors:** Paraskevas Zafeiropoulos, Andreas Katsanos, George Kitsos, Maria Stefaniotou, Ioannis Asproudis

**Affiliations:** University Ophthalmology Clinic, Stavros Niarchos Avenue, 45500 Ioannina, Greece

**Keywords:** Multiple sclerosis (MS), Multifocal visual evoked potential (mfVEP), Optic neuritis (ON)

## Abstract

**Purpose:**

To review the evidence on the usefulness of the multifocal visual evoked potential (mfVEP) test in patients with optic neuritis (ON) and/or multiple sclerosis (MS).

**Methods:**

We critically review key published evidence on the use of mfVEP in ON/MS patients and its association with other functional and structural tests.

**Results:**

Multifocal VEP tests are useful in detecting abnormality in patients with ON/MS and monitor the progression of lesions (remyelination, atrophy). In addition, mfVEP has good correlation with conventional visual evoked potential (VEP), standard automated perimetry, optical coherence tomography and magnetic resonance imaging. In patients with ON, mfVEP might be useful in predicting the risk of conversion to MS.

## Introduction

Multiple sclerosis (MS) is an autoimmune, demyelinating, neurodegenerative disease of the central nervous system (CNS) [1]. Optic neuritis (ON) is a type of inflammatory demyelination of the CNS and is often the presenting symptom in approximately 25% of MS patients. For the majority of patients, the disease has a relapsing–remitting course [2]. A single demyelinating episode of unknown, possibly viral etiology, is called Clinically Isolated Syndrome (CIS) [3]. More than 80% of CIS patients with lesions on magnetic resonance imaging (MRI) will develop MS [4]. In patients with MS, the visual pathway can be involved along its entire course from the outer retina to the visual cortex [5].

Using scalp electrodes, the visual evoked potential (VEP) is a gross electrical potential recorded, using scalp electrodes, from the visual cortex [6]. mfVEP is a relatively new objective test [7] for evaluating the integrity of the visual pathway [8]. mfVEP combines visual evoked potential recordings in response to a dartboard-like pattern stimulus display that is subdivided into a number of sectors (up to 60) each with several checks, which covers over 40 degrees of the visual field. Responses to a pseudo-random sequence of contrast reversal for each region are combined in a continuous electroencephalography (EEG) signal recorded around the inion. The software, using a sophisticated mathematical algorithm, extracts 60 mfVEP responses, each associated with one sector of the display [9, 10].

### Technical aspects of mfVEP testing

In mfVEP responses, both amplitude reduction and latency delay are estimated. Besides amplitude and latency of the responses of one eye (monocular amplitude and monocular latency, respectively), the difference of amplitude or latency between the two eyes of a patient can be estimated (interocular amplitude or interocular latency). The amplitude is usually expressed as signal-to-noise ratio (SNR). SNR is the root mean square (RMS) of the sector’s waveform in an interval of 45–150 ms (signal window) divided by the mean RMS in an interval of 325–430 ms (noise window) of all 60 sectors [6, 10, 11]. Abnormal responses may be expressed as:

I. Mean amplitude or latency
II. Probability plots, which are maps of defects similar to the Humphrey visual field defect maps.
III. Clusters of adjacent abnormal sectors in a map of the 60 sectors of mfVEP.

Localized defects are more readily revealed with interocular comparison of the mfVEP (interocular test). To obtain an interocular mfVEP probability plot, the ratio of the amplitudes of the mfVEP of the two eyes is measured for each sector of the display. This ratio is then compared to the ratios from a group of controls to establish 5% and 1% significance levels.

In MS patients, factors such as attention deficits, fatigue and transient worsening of symptoms due to changes in core body temperature (Uhthoff’s phenomenon) may potentially influence the outcome of a test [12]. Three limitations of mfVEP should be noted [9]:

i. Spatial resolution in the periphery is relatively poor, as sectors in these locations subtend areas over 7° in width. Considering that significant change should be found in at least two contiguous areas in order to reliably confirm damage, it becomes evident that relatively large peripheral defects may be overlooked.
ii. Patients who cannot cooperate or fall asleep or cannot open their eyelids sufficiently cannot perform the test reliably.
iii. Eccentric fixation produces unreliable results.

Currently, the two most widely used mfVEP platforms are the original VERISScientific™ system (Electro-Diagnostic Imaging, Redwood city, CA, USA) and the AccuMap™ system (ObjectiVision, Sydney, Australia).

### Evidence for the usefulness of mfVEP in ON/MS

mfVEP testing can be a valuable option in the following clinical scenarios: as a means of ruling out non-organic visual loss, as a tool for diagnosing ON, for the follow-up of patients with known ON/MS, and lastly, for the evaluation of patients with unreliable or questionable perimetry [9]. In a multicenter study, mfVEP has also been used as an endpoint for candidate CNS neuroreparative treatments in acute optic neuritis [13]. Table 1 summarizes key available evidence on the usefulness of mfVEP in patients with ON/MS.

**Table 1:** Aims and primary outcomes of studies employing mfVEP in patients with optic neuritis and multiple sclerosis

References	Patients	Tests	Aim	Primary outcome
Narayanan et al. [12]	40 RRMS patients (25 ON eyes 34 non-ON eyes) 40 controls	mfVEP, VEP	To establish reproducibility of mfVEP and traditional VEP in controls and RRMS patients	mfVEP and VEP showed good reproducibility in controls and RRMS patients
Fortune et al. [14]	50 controls	mfVEP	To assess the repeatability of mfVEP and compare with repeatability of SAP	Good reproducibility of mfVEP amplitude across eyes/locations, which was slightly better than standard automated perimetry
Fraser et al. [15]	64 patients with ON	mfVEP	To determine the sensitivity of mfVEP in optic neuritis	The authors concluded that mfVEP is a sensitive and specific tool for detecting ON
Fraser et al. [16]	46 patients with ON	mfVEP	To monitor the difference in conversion rates to MS in patients with mfVEP latency delay and those with normal latency	mfVEP latency may assist in predicting progression to MS
Pakrou et al. [17]	16 patients with ON	mfVEP, HVF	To compare mfVEP with HVF in the assessment of patients with ON	The mfVEP detected more abnormalities in patients with ON compared with HVF
Groover et al. [18]	19 patients with ON/MS	mfVEP, VEP	To compare VEP and mfVEP in patients with ON/MS	The mfVEP was superior to VEP in detecting ON/MS
Klistorner et al. [19]	26 patients with ON	mfVEP, VEP	To compare VEP and mfVEP in patients with ON	mfVEP was superior to detect abnormalities in the periphery or the upper hemifield
Klistorner et al. [20]	48 patients with ON	mfVEP	To investigate the electrophysiological changes in fellow eyes of patients with a single episode of ON	Association between the risk of MS and the magnitude of amplitude reduction and latency prolongation was found
Klistorner et al. [21]	50 patients with ON	mfVEP, OCT	To investigate relationship between mfVEP and OCT	Strong topographical associations between mfVEP and OCT
Laron et al. [8]	69 patients with MS	mfVEP, OCT, HVF	To compare mfVEP, OCT, HVF	mfVEP reveals more abnormalities than OCT and HVF
Alshowaeir et al. [5]	59 patients with MS	mfVEP, MRI	To prove that the latency delay of mfVEP in non-ON eyes of MS patients is related to retrochiasmal lesions	The latency delay of mfVEP in non-ON eyes of MS patients is related to retrogenicular demyelinating lesions
Blanco et al. [22]	28 patients with MS and ON	mfVEP, OCT, HVF, EDSS score	To evaluate visual function and relationship between disability and ON in patients with MS	Significant relationship between mfVEP amplitude and disease severity
Pérez-Rico et al. [23]	29 patients with CIS	mfVEP, OCT, HVF	To evaluate visual pathway in patients with CIS	Combined use of OCT and mfVEP reveals subclinical abnormalities and axonal loss in CIS patients
Sriram et al. [24]	58 patients with MS	mfVEP, ERG, OCT	To investigate the relationship between the visual acuity and electro physiological tests with RGC	There is significant association of RGC decrease in non-ON eyes of MS patients with retinal dysfunction and post-chiasmal damage
Alshowaeir et al. [25]	87 patients with ON	mfVEP	To evaluate mfVEP changes in ON and fellow eye during the first year of attack	mfVEP amplitude early predicts post-ON axonal loss
Van der Walt et al. [26]	30 patients with ON	mfVEP, MRI	To investigate the relationship between mfVEP latency and optic nerve lesion after acute ON	There was strong association between mfVEP and MRI findings for demyelination in acute and chronic ON
Sriram et al. [24]	40 patients with MS	mfVEP, VEP	To prove reproducibility of mf VEP	mfVEP showed good reproducibility in normal and MS patients
De Santiago et al. [11]	71 patients with MS	mfVEP	Use of SNR of mfVEP to estimate the risk of developing MS	SNR analysis of mfVEP amplitude may estimate the risk to develop MS
Shen et al. [27]	136 patients with MS and 19 patients of NMOSD	mfVEP, OCT, MRI	To investigate the differences of axonal loss and demyelination in MS and NMOSD	Different patterns of damage in NMOSD and MS revealed. The cause of ON damage was in MS demyelination Across the visual pathway was the cause of ON damage, but in NMOSD was axonal damage in the anterior visual pathway
Narayanan et al. [28]	90 patients with MS	mfVEP, OCT, HVF, contrast sensitivity	To evaluate the relationship between structural and functional tests in eyes of MS patients	mfVEP and CS had good correlation with structural measurements. mfVEP revealed more abnormalities in non-ON eyes than OCT
Klistorner et al. [13]	39 (48%) participants from the RENEW study	mfVEP, VEP	mfVEP testing was used to study changes in visual pathways of 48% of participants that had suffered their first acute unilateral ON episode and were recruited in the placebo-controlled RENEW trial	From this substudy, advantages of mfVEP over VEP were revealed in participants of a multicenter trial examining CNS reparative therapies. It was shown that fellow eye visual pathway amplitude loss occurs after ON, but this can potentially be prevented by opicinumab treatment

*CIS* clinical isolated syndrome, *CNS* central nervous system, *CS* contrast sensitivity, *EDSS score* Expanded Disability Status Scale score, *ERG* electroretinogram, *HVF* Humphrey visual field, *mfVEP* multifocal visual evoked potentials, *MS* multiple sclerosis, *NMOSD* neuromyelitis optica spectrum disorders, *OCT* optical coherence tomography, *ON* optic neuritis, *RGC* retinal ganglion cells, *RRMS* relapsing–remitting multiple sclerosis, *SAP* standard automated perimetry, *SNR* signal-to-noise ratio, *VEP* visual evoked potential

For the mfVEP to detect and monitor MS-related changes, it is crucial to define its reproducibility. Fortune et al. [14] reported good reproducibility of mfVEP amplitude across eyes/locations, which was slightly better than standard automated perimetry, in a group of 50 normal controls. Narayanan et al. [12] assessed the reproducibility of mfVEP and traditional pattern-reversal VEP amplitude and latency in the eyes of patients with relapsing–remitting multiple sclerosis MS (RRMS) who suffered their last ON event at least 6 months previously (ON group, *n* = 25), eyes of RRMS patients without a history of ON (non-ON group, *n* = 34) and eyes of age-matched controls (*n* = 40). Reproducibility was assessed using two different methods: intraclass correlation coefficient (ICC) and test–retest variability (TRV). Mean mfVEP global amplitude was reduced for the non-ON and ON groups compared to controls. Mean global latency was delayed in the ON and non-ON groups compared to controls. There was good intervisit agreement amplitude and latency in normal, non-ON and ON eyes. Reproducibility of mfVEP amplitude was similar across all regions and groups and did not depend on magnitude of amplitude. Good reproducibility of mfVEP latency was observed in all regions and groups with high ICC values, all above 0.80 (ICC ≥ 0.75 is typically considered “good”). These authors also found that mfVEP latency variability, as expressed with TRV, was similar in normal, non-ON and ON eyes, while ON eyes showed greater latency variability than non-ON and normal eyes. These findings support the notion that subclinical changes occur in eyes with ON within a short time interval. In general, mfVEP and conventional VEP showed good reproducibility of amplitude and latency in normal and RRMS eyes. To our knowledge, this is the only study characterizing the reproducibility of mfVEP in MS patients and controls.

## Multifocal VEP results in diagnosis and evaluation of ON and MS patients

mfVEP is a test with good sensitivity and specificity in detecting visual pathway abnormalities. In patients with ON and MS, amplitudes are decreased and latencies are delayed. Latency delay is a very important finding when patients are being evaluated for possible MS, because it points toward the hallmark of demyelinating diseases, i.e., demyelination-induced delay in signal conduction. Abnormal latency measurements can thus contribute to the diagnosis of subclinical demyelination. Decreased amplitude suggests neural degeneration and signifies axonal loss in patients with MS [9].

The characteristics of mfVEP studies in patients with ON and MS are summarized in Table 1.

Using the AccuMap system, Freser et al. [15] examined 64 patients with inflammatory or demyelinating ON. They classified them into three groups using the McDonald criteria: a non-MS group (27 eyes), a possible MS group (25 eyes), a definite MS group (24 eyes) compared with a control group (20 eyes). The majority of cases in all three groups had an amplitude abnormality. Specifically, amplitude abnormalities were detected in 92.6% of eyes in the non-MS group, 92.0% of eyes in the possible MS group and 100% of eyes in the definite MS group. Regarding latency, there was significant difference between the rates of latency abnormality for each of three groups: 100% for the MS group, 76% for the possible MS group and 33.3% for the non-MS group. The authors concluded that mfVEP is a sensitive and specific tool for detecting ON. In a different study, the same investigators [16] performed mfVEP in 46 of these patients with ON who were not diagnosed with MS. The analysis showed that only 22 subjects exhibited mfVEP delay. Over a period of 1 year, 36.4% of patients with ON with latency delay progressed clinically to MS compared with 0% of those with normal latencies. This may indicate that mfVEP latency delay can assist in predicting progression to MS.

A possible predictive role of mfVEP was revealed in a study that investigated the electrophysiological changes in fellow eyes of patients with a single episode of ON and no previous demyelination 12 months after the episode. Klistorner et al. [20] found an association between the risk of MS and the magnitude of amplitude reduction and latency prolongation.

Recently, Alshowaeir et al. [25] evaluated mfVEP changes in ON and fellow eyes during the first year after the attack. They examined 87 patients with clinically diagnosed typical acute unilateral ON (27 of 87 patients were considered low risk, and 60 of 87 patients were considered high risk for developing MS) and 25 healthy controls. mfVEP recordings were performed at 1, 3, 6 and 12 months after the attack of ON. Their results indicate that both amplitude and latency of the mfVEP are grossly abnormal during the early stage of ON. Following an episode of ON, the recovery of mfVEP amplitude and the shortening of mfVEP latency are fastest within the first 3 months. There is significant residual latency delay even 12 months after the attack. This chronically persisting latency delay has been demonstrated in multiple studies and remains the major hallmark of previous ON episodes. The severity of amplitude reduction and latency delay after an episode of ON is not MS related, despite the fact that differences in amplitude and latency between MS and non-MS patients have been found. These differences are attributed to retrochiasmal demyelination in MS patients.

De Santiago et al. [11] examined the variation in mfVEP amplitude, quantified as SNR, across six concentric rings of the visual field. These concentric rings are of increasing retinal eccentricity from 1° (for the most central ring) to 22.2°. The authors analyzed three groups: patients with Radiologically Isolated Syndrome (RIS, *n* = 15), patients with Clinically Isolated Syndrome (CIS, *n* = 28) and patients with definite MS (*n* = 28). As for optic neuritis (ON) subjects’ eyes, these were classified as ON affected or non-ON affected. The control group was composed of 24 age-matched healthy participants. As expected, a significant reduction of mfVEP amplitudes SNR values was observed in clinically definite MS patients when compared with the control group (SNR control: 0.70, SNR MS–non-ON: 0.52, SNR MS–ON: 0.47). No statistically significant difference was observed between MS–ON and MS–non-ON eyes, because most non-ON eyes have been shown to be sub-clinically affected in clinically definite MS. The authors concluded that SNR values of mfVEP amplitude steadily decrease, especially in rings 3 and 5, as MS risk increases. Due to the fact that MS affects the visual pathway early in the course of the disease, this SNR analysis could be used to identify the risk of MS progression. Additionally, the results of this study showed a significant association between mfVEP amplitude and severity of disability in the Expanded Disability Status Scale (EDSS) in clinically definite MS.

## Comparison with other structural or functional diagnostic techniques

As discussed later in this article, compared to the conventional full-field VEP, the mfVEP has shown superior sensitivity and specificity in most studies. Conventional full-field VEP provides a summed response of all stimulated neuronal elements and is greatly dominated by the macular region due to the cortical overrepresentation of the latter. Conventional VEP is prone to unpredictable changes depending on the part of the nerve/visual field affected. This can lead to detection of apparent, rather than real latency delay and waveform distortion. In 26 subjects with a history of unilateral ON and no previous diagnosis of MS, the amplitude of the affected eye was statistically significantly decreased compared to the fellow eye for both conventional and mfVEP. There was also statistically significant difference for the latency between the patients’ two eyes. Compared to conventional VEP, mfVEP was superior in detecting amplitude and latency abnormalities when the affected area was located in the periphery or the upper hemifield [19].

To examine the potential advantages of mfVEP compared to conventional VEP, Grover et al. [18] employed the two methods in 19 ON/MS patients and 40 controls. The best performance of mfVEP in detecting damage secondary to ON/MS was observed when the interocular and monocular tests were combined. In this case, mfVEP had a sensitivity of 94.7% and a specificity of 90%. In conclusion, the mfVEP was superior to conventional VEP in detecting ON/MS, but the difference was less than expected (mfVEP detected only two more patients compared to conventional VEP).

Compared to automated perimetry, mfVEP seems capable to detect more abnormalities. There is also good topographical agreement between mfVEP and standard automated perimetry. Pakrou et al. [17] compared the use of mfVEP versus Humphrey visual fields (HVFs) in patients with ON. A total of 25 affected eyes and seven non-affected eyes, from 16 patients with a history of ON, underwent mfVEP and HVF. Field test results of each eye were divided into four quadrants. The presence or absence of scotoma for each sector was identified for HVF fields and for mfVEP from both amplitude plots and mfVEP latency clusters. A total of 128 mfVEP amplitude and latency quadrants (100 in affected and 28 in unaffected eyes) were analyzed and compared topographically with the quadrants from HVF. HVF was able to identify scotomas in 30.5% of quadrants, all from affected eyes. On the other hand, mfVEP was able to identify scotomas in 57.8% of quadrants, but three of the quadrants were from clinically unaffected eyes. There was a 95% agreement between mfVEP and HVF in identifying a scotoma. Seventy-five percent of total eyes and 80% of the affected eyes had significant latency deviation. Additionally, abnormalities in the clinically unaffected eye were detected in 67% of cases. This study suggests that mfVEP may be useful in patients with ON and may also be more sensitive to detect subtle defects or defects in the clinically unaffected eye. Furthermore, latency recordings in combination with amplitude and asymmetry plots detect abnormalities otherwise undetected with standard automated perimetry.

The correlation between mfVEP and optical coherence tomography (OCT) has been investigated. Klistorner et al. [21] examined the topographical correspondence between OCT-derived RNFL thickness and mfVEP amplitude. The authors examined 50 patients who suffered an attack of acute unilateral ON at least 6 months previously and 25 age-matched controls. Superior, temporal and inferior sectors were compared. The nasal sector of RNFL corresponds to only 4 of 60 sectors of mfVEP testing (two of them include the blind spot) and was therefore not analyzed. Thirteen of the patients were diagnosed with MS, and 37 had ON as CIS. There was statistically significant reduction in the affected eyes in the superior, temporal and inferior sectors of RNFL (20%, 25% and 21%, respectively) compared to controls. In addition, mfVEP amplitude was significantly reduced by 34%, 44% and 37% for corresponding parts of the visual field. The largest reduction was observed in the temporal sector of the RNFL and the mfVEP amplitude at the corresponding central part of the field. In eyes with ON, the amplitude of the mfVEP was disproportionately reduced compared to the RNFL thickness, possibly due to subclinical inflammation of the visual pathway which may affect mfVEP amplitude before axonal loss becomes detectable by OCT.

Laron et al. [8] performed mfVEP, HVF and OCT in 69 patients with clinically definite MS. Their aim was to compare the sensitivity of mfVEP testing versus the sensitivity of HVF and OCT in detecting abnormalities in MS patients. The authors used two study groups: One included 47 MS–ON eyes (latest ON attack at least 6 months previously) and the other included 65 eyes without a history of ON (MS–non-ON group). For all parameters, there were statistically significant differences between the MS–ON group and the MS–non-ON group. In the MS–non-ON group, more eyes were found to be abnormal based on HVF (38%) or mfVEP using both amplitude and latency (amp/lat) information (29%), than OCT (8%). Amplitude alone, calculated as logSNR, revealed abnormality in 15% of MS–non-ON eyes and latency in 25% of MS–non-ON eyes. Both mfVEP and HVF detect abnormality in 18% of eyes of the MS–non-ON group. This suggests that about 20–40% of MS patient’s eyes without evident episode of ON have had a subclinical episode in the visual pathway. mfVEP (amplitude/latency probability plots meeting cluster criteria with 95% specificity) identified more abnormality in MS–ON eyes (89%) than HVF (72%), OCT (62%), mfVEP amplitude (66%) or latency (67%) alone. The results of this study suggest that mfVEP performed better compared to HVF and OCT because of its ability to detect demyelination as latency abnormalities. Topographical agreement between these tests ranged from 60 to 79%.

To evaluate the relationship between optic nerve dysfunction and disability in patients with MS and ON using mfVEP, Blanco et al. [22] examined 28 patients with clinically definite MS and an ON episode at least 6 months prior to study recruitment. The authors used three groups: one that included 37 eyes of these patients with MS-related ON (MS–ON group), one that included their 19 fellow eyes without a history of ON (MS–non-ON group), and one group composed of one eye from each of 19 age-matched healthy controls. All participants underwent HVF, OCT and mfVEP. Disability was assessed using the Expanded Disability Status Scale (EDSS) score. HVF mean deviation (MD) and pattern standard deviation (PSD) revealed significant differences between all groups. Also, 81% of the eyes in the MS–ON group and 84% of the eyes in the MS–non-ON group showed amplitude and/or latency defects in the mfVEP, but no statistically significant difference between the MS–ON and MS–non-ON groups was revealed. The EDSS score was significant different (*p* = 0.019) between patients with normal and abnormal mfVEP, suggesting a relationship between the extent of axonal loss and neurological impairment in MS patients.

Patients with CIS are at risk to develop MS. Although MRI imaging findings are the best known predictive factors, visual pathway involvement revealed by OCT and mfVEP may have a predictive value. Using mfVEP, HVF and OCT, Pérez-Rico et al. [23] tested the eyes of 29 consecutive patients with CIS. The study group was made up from one randomly selected eye of 20 CIS patients without ON history and the unaffected eye of nine CIS patients with unilateral ON. The control group consisted of 26 eyes randomly selected from each of 26 healthy participants. HVF indices did not show significant differences between the CIS and the control eyes. As for mfVEP, 59% of CIS eyes revealed amplitude and/or latency defects. mfVEP amplitude and latency responses of the unaffected eyes were abnormal. A significant proportion of CIS patients (65.5%) converted to MS according to the McDonald criteria within 12 months. Besides, the average RNFLT at baseline was found to have some statistically predictive value for MS conversion. In conclusion, subclinical optic nerve involvement was detected in CIS eyes using mfVEP and OCT.

The ganglion cell layer (GCL) consists of retinal ganglion cells and, because of its position, is easily accessible to observation by high-resolution spectral domain OCT. Using OCT, mfVEP and high-resolution MRI, Sriram et al. [24] assessed 58 non-ON eyes of 58 MS patients, 25 of whom had a history of optic neuritis episode to the other eye. The authors reported significant RNFL and GCL thinning in non-ON eyes of MS patients compared to controls (*p* = 0.002 and *p* < 0.0001, respectively). They also reported significant correlations of RNFL and GCL thickness with amplitude reduction of the mfVEP. In addition, a comparison between the study eye and the fellow eye in patients with no episode of ON in any eye revealed a significant correlation in RNFL and GCL thinning. In addition, mfVEP latency had a similar pattern of binocular delay and exhibited a significant correlation with RNFL and RGC thinning. These results in combination with the fact that ON fibers are partially crossing at the chiasm may constitute evidence of retro-chiasmal nerve damage and trans-neuronal degeneration caused by lesions in the optic radiation.

Recently, Narayanan et al. [28] examined the relationship between structural tests such as ganglion cell inner plexiform layer (GCIPLT) and functional test such as mfVEP amplitude and latency, Pelli–Robson contrast sensitivity (CS) and HVF in eyes with or without a history of ON involvement in RRMS patients. Ninety patients were enrolled in the study, and data from 105 MS eyes with no history of ON (non-ON eyes) and 53 eyes with last episode of ON at least 6 months previously were analyzed. All mean values of all tests were worse in ON than non-ON or normal eyes (MD: *p* = 0.02; all other comparisons: *p* < 0.0001). In non-ON eyes, mean values were worse than their respective normative values for GCIPLT, MD, mfVEP amplitude and mfVEP latency. Using Pearson correlation, in ON eyes all measures from functional tests significantly correlated with GCIPLT (*r* = − 0.40 to 0.78; *p* = 0.03 to 0.0001). In non-ON eyes, all measures from functional tests except HVF significantly correlated with GCIPLT (*r* = − 0.24 to 0.51; *p* = 0.04 to 0.0001). In non-ON eyes, mfVEP performed significantly better than OCT and detected 23% more abnormal eyes than OCT.

Neuromyelitis optica spectrum disorders (NMOSD) and MS are both idiopathic, autoimmune, inflammatory disorders of CNS with common clinical manifestations. In a study with 136 MS patients, 19 patients suffering from NMOSD and 37 healthy participants, Shen et al. [27] performed mfVEP, OCT and MRI. In ON eyes from MS and NMOSD patients, significant differences were revealed in RNFL, GCIPL, mfVEP amplitude and mfVEP latency compared with those of controls. In non-ON eyes of MS patients, significant differences compared to controls were detected for RNFL and GCIPL. In addition, non-ON eyes of MS patients had significantly different values in mfVEP amplitude and latency compared to controls. There were no significant differences, for non-ON eyes between NMOSD patients and controls for RNFL, GCIPL, mfVEP amplitude and latency. For ON eyes, there were significant correlations between mfVEP and OCT parameters in both the MS and the NMOSD groups. As for non-ON eyes, correlations between mfVEP and OCT were found only for the MS patients. Delayed latency of mfVEP and thinning of RNFL in non-ON eyes of MS patients may have been caused by demyelinating lesions in the optic radiation, as such findings have been documented at this part of the optic tract using diffusion tensor magnetic resonance imaging (MRI). A key finding of this study is that a different pattern of optic nerve damage occurs in MS and NMOSD patients with ON. In NMOSD, there is more severe axonal loss, which is apparently related to ON attacks. In ON eyes of NMOSD patients, there is reduction in RNFL and GCIPL thickness, along with reduced mfVEP amplitude. There are no significant losses of RNFL and GCIPL and no significant reduction of mfVEP amplitude in non-ON eyes of NMOSD patients. In MS patients, demyelination seems to be the pathomechanism underlying the reduction of RNFL and GCIPL thickness and the prolongation of mfVEP latency in eyes with and without known previous ON attacks. These findings had significant relationship with the volume of optic radiation lesions as displayed in MRI [27].

To test the hypothesis that mfVEP latency delay in non-ON eyes of MS patients is related to retrochiasmal demyelinating lesions, Alshowaeir et al. [5] used mfVEP and MRI in 59 patients with RRMS and no history of ON in at least one eye. The patients’ data were compared with those of 25 healthy controls. The mfVEP latency in non-ON eyes was significantly delayed compared to controls. Optic radiation lesions were revealed in 77% of the patients. There were significant associations between mfVEP latency and optic radiation T2 FLAIR lesion load. Two subgroups were studied: a group of fellow (unaffected) eyes of patients with unilateral ON and a group of eyes from patients without history of ON in any eye. Significant correlation between mfVEP latency and optic radiation lesion volume was found only in the latter group. The investigators concluded that optic radiation lesions are associated with latency delay in non-ON eyes of MS patients.

Van der Walt et al. [26] examined the correlation between optic nerve lesion length and mfVEP latency delay in a cohort of 30 patients with a recent first episode of unilateral ON. The patients underwent a cerebral MRI scan and mfVEP testing. The patients were examined 2 weeks after symptom onset and then after 1, 3, 6 and 12 months. At the end of the first month of follow-up, the mfVEP amplitude had improved in 23 (76%) of patients. Significant improvement in latency was noted during the follow-up period. Recovery of mfVEP latency was fastest during the first 2 months of the follow-up period. A single lesion, using a 3D T2-weighted sequence, was identified in 20 of 23 (87%) affected optic nerves. The length of the T2 lesion diminished significantly during the follow-up period. The speed of T2 lesion shortening was similar during the entire follow-up period. There was a significant correlation between lesion length asymmetry and mfVEP latency asymmetry during the follow-up period.

In a recent study, Klistorner et al. [13] used mfVEP to study changes in the visual pathway of 48% of the participants included in the RENEW trial [29]. The aim of the placebo-controlled RENEW trial was to investigate opicinumab (a fully human monoclonal antibody against LINGO-1), as a treatment of CNS demyelinating disorders in participants after a first episode of unilateral acute ON [29]. In the study by Klistorner et al. [13], changes in latency and amplitude in the affected and fellow eyes of patients were measured using mfVEP and conventional full-field VEP. Both groups showed mfVEP latency prolongation at the end of treatment (24 weeks) and at the end of study (week 32). In both treatment groups, there was mfVEP amplitude recovery from baseline in the affected eye. A post hoc comparison of estimated effect size for change in mfVEP and conventional VEP latency for opicinumab versus placebo at week 24 showed that mfVEP demonstrated a larger treatment effect size than conventional VEP. This indicates that mfVEP may be superior to conventional VEP in highlighting similar treatment effects on latency delay with a smaller sample size. There was no change in mfVEP latency and amplitude in fellow eyes in both treatment groups. On the other hand, there was significant loss of mfVEP amplitude in the placebo group. This study [13] was the first in which mfVEP was employed as an outcome measure in a multicenter therapeutic trial. In addition, it established the feasibility of using mfVEP as a biomarker for candidate CNS neuroreparative treatment studies in acute ON.

## Conclusion

mfVEP is a relatively new objective functional test of the optic tract. It contributes to the diagnosis, monitoring and risk assessment of ON/MS patients, especially when combined with other functional and structural tests of the optic path. mfVEP has the potential to reveal subclinical lesions in patients with MS with a negative history for ON. By detecting latency abnormalities, mfVEP can direct clinical diagnostic algorithms toward demyelinating processes. This data could help determine the effect of possible therapeutic interventions in the future.

However, mfVEP testing has certain limitations. It is a time-consuming procedure that must be performed by meticulous, well-trained technicians and be interpreted by knowledgeable assessors. Commercial software for adequately analyzing the mfVEP is not yet available, and the analysis of results has yet to be standardized. Reasonably large defects in the outer ring might be missed because spatial resolution can be quite poor in the periphery and eccentric fixation may affect the results. Although mfVEP is a promising method for research and clinical purposes, standardization and further refinements in software are expected to improve the utility of the technique in the future.
